# miR‐129‐2‐3p directly targets SYK gene and associates with the risk of ischaemic stroke in a Chinese population

**DOI:** 10.1111/jcmm.13901

**Published:** 2018-11-29

**Authors:** Suli Huang, Ziquan Lv, Ying Wen, Yazhen Wei, Li Zhou, Yuebin Ke, Yanwei Zhang, Qianhui Xu, Lu Li, Yinsheng Guo, Di Li, Changhui Xie, Yi Guo, Jinquan Cheng

**Affiliations:** ^1^ Department of Molecular Epidemiology Shenzhen Center for Disease Control and Prevention Shenzhen Guangdong China; ^2^ Department of School Hygiene Shenzhen Center for Disease Control and Prevention Shenzhen Guangdong China; ^3^ Department of Neurology People's Hospital of Shenzhen Guangdong China; ^4^ Research Center of Translational Medicine The Second Affiliated Hospital of Shantou University Medical College Shantou Guangdong China

**Keywords:** ischaemic stroke, miR‐129‐2‐3p, platelet, spleen tyrosine kinase

## Abstract

Spleen tyrosine kinase (SYK) gene has been identified as novel susceptibility locus for ischaemic stroke (IS) previously. However, regulation of SYK gene remains unknown in IS. In this study, we aimed to identify miRNAs that might be involved in the development of IS by targeting SYK gene. miRNAs were firstly screened by bioinformatics predicting tool. The expression levels of SYK gene were detected by qRT‐PCR and western blotting, respectively, after miRNA transfection. Luciferase reporter assay was applied to investigate the direct binding between miRNAs and target gene. miRNA levels were detected by miRNA TaqMan assays in the blood cells of 270 IS patients and 270 control volunteers. Results suggest that SYK gene might be a direct target of miR‐129‐2‐3p. The blood level of miR‐129‐2‐3p was significantly lower in IS patients (*P* < 0.05), and negatively associated with the risk of IS (adjusted OR: 0.88; 95% CI: 0.80‐0.98; *P* = 0.021) by multivariable logistic regression analysis. The blood levels of SYK gene were significantly higher in IS patients, and miR‐129‐2‐3p expression was negatively correlated with mean platelet volume. In summary, our study suggests that miR‐129‐2‐3p might be involved in the pathogenesis of IS through interrupting SYK expression and the platelet function, and further investigation is needed to explore the underlying mechanism.

## 1 INTRODUCTION

Stroke is the second leading cause of death among people over 60 years old worldwide.^1^ According to the World Health Organization, 15 million people suffer stroke globally each year. In China, ischaemic stroke (IS) is the most common type of stroke, accounting for 43%‐79% of the total number of stroke.^2^ Cerebrovascular atherosclerosis is the major pathogenic mechanism of IS, which entails chronic inflammation to arterial vessel walls and cross‐talk with procoagulant signaling pathways, culminating in plaque rupture and thrombosis.^3^, ^4^ In addition to traditional risk factors, such as age, male sex, smoking, history of hypertension, diabetes mellitus and atrial fibrillation, amounting genetic risk factors have been identified in recent years by genome‐wide association studies and exome sequencing.^5^, ^6^, ^7^, ^8^, ^9^, ^10^

We have previously identified several susceptibility loci for IS in a Chinese Han population.^11^ Exome sequencing was used to screen susceptibility loci among 100 cases and 100 matched controls. Significant SNPs were further verified in up to 3554 participants from three hospital‐based case‐control studies. Ultimately, we identified two novel loci (rs17118 of XYLB gene located on chromosome 3p21, and rs10489177 of c1orf156 gene located on chromosome 1q24), which were associated with an increased risk of IS. Besides, we also found that the frequency of rs2290890 T allele of spleen tyrosine kinase (SYK) gene was associated with a decreased risk of IS (data not published). SYK gene, located on chromosome 9p22 and encoding a non‐receptor type of protein‐tyrosine kinase, that is, widely expressed in hematopoietic cells, is one of the two members of the Syk family (Syk and ZAP‐70), and has been extensively studied as a critical regulator in the pathogenesis of cancer.^12^ SYK gene polymorphisms have been also reported to be associated with the risk of non‐Hodgkin lymphoma^13^ and prostate cancer.^14^ Recently, the role of SYK in vascular inflammation and atherosclerosis has attracted great interest. In the low‐density lipoprotein receptor‐deficient mice consuming a high‐cholesterol diet supplemented with orally available SYK inhibitor fostamatinib, the atherosclerotic lesion size was reduced by 59%, the adhesion and migration of inflammatory cells were attenuated, and macrophage survival was limited, suggesting SYK inhibition as a potentially fruitful anti‐inflammatory therapeutic strategy in atherosclerosis.^15^ In addition, Bijli et al suggested that Syk signals downstream of PKC‐delta to mediate thrombin induced ICAM‐1 expression in endothelial cells by increasing transcriptional capacity of NF‐kappaB via a mechanism that relies on tyrosine phosphorylation of RelA/p65.^16^ According to the literature review and our previous findings, we supposed that the dysregulation of SYK expression might be crucial for the development of IS, an atherosclerosis‐related disease. However, no direct evidence has been reported about the expression and regulation of SYK in IS.

miRNAs are a class of 22‐ nucleotide‐ long endogenous non‐coding RNAs which participate in the post‐transcriptional regulation of genes by binding to the 3′ untranslated region (3′UTR). miRNAs are well recognized as key regulators in the pathological events of IS, including atherosclerosis,^17^ hyperlipidemia,^18^ hypertension^19^ and plaque rupture.^20^ Inspired by the clinical application of oral SYK inhibitors as previously indicated,^15^ we further have been suggested that the decrease in SYK expression might be partially regulated by miRNA. To figure out the miRNAs that might target SYK expression and the miRNAs with potential clinical value as biomarker for IS, we firstly screened for miRNAs by the bioinformatics predicting tool TargetScan 7.1. The miRNA expression profiles of the whole blood cells of 25 IS patients and 25 control volunteers were examined by miRNA microarray. Results suggest that 455 miRNAs were differentially expressed (*P* < 0.05),^21^ among which 20 miRNAs with top cumulative weighted context score, as indicated by TargetScan 7.1, were ultimately selected for further investigation. Regulation of SYK expression by miRNAs was first examined by cell experiments. The target miRNA was then detected in an independent case‐control population composed of 270 IS patients and 270 age‐sex matched control volunteers. The potential value of the miRNAs for IS risk prediction was analysed by multivariate logistic regression.

## 2 MATERIALS AND METHODS

### 2.1 Study population

The whole blood cell levels of miR‐129‐2‐3p were detected in a case‐control population composed of 270 IS patients and 270 control volunteers. The patients were recruited from Shenzhen People's Hospital between June 2014 and December 2016. All cases were inpatients diagnosed with IS for the first time and were recruited based on the appearance of a new and abrupt focal neurological deficit, with neurological symptoms and signs persisting for more than 24 hours, as described previously.^11^, ^21^ IS was confirmed by positive findings on head CT or MRI according to the International Classification of Disease (9th Revision, codes 430 to 438). Patients with a history of stroke, peripheral arterial occlusive disease or cancer were excluded from this study. Participants without medical history of cerebrovascular diseases were selected as controls during a physical examination at the eighth affiliated hospital of Sun Yat‐Sen University from August 2014 and May 2016, and were matched with patients by age and sex. Approximately 5 mL of vein blood samples were collected from each participant in EDTA‐anticoagulant tubes and stored at −80°C until use. Blood samples from patients with IS were collected within 12 hours after hospital admission. The small patient cohort for SYK expression detection was randomly selected from the whole population.

For all participants, structured questionnaires were used by trained interviewers to collect information about demographic characteristics and clinical biochemistry. The ethics committee of the Shenzhen Center for Disease Control and Prevention approved this study, and written informed consent was obtained from each participant. The research was carried out according to the World Medical Association Declaration of Helsinki.

### 2.2 Total RNA isolation

Approximately 200 μL of blood cells/sample was applied to isolate the total RNAs using the mirVana PARIS miRNA Isolation Kit (Ambion 1556, Austin, TX, USA) according to the manufacturer's protocol with slight modifications, namely, each sample was treated twice with acid‐phenol chloroform. The total RNAs of cultured cells were isolated using Trizol reagent (Invitrogen, Carlsbad, CA, USA). For the detection of *SYK* gene expression, the total RNA was isolated from 500 μL of whole blood cells/sample using Trizol LS reagent (Invitrogen) according to the manufacturer's protocol.

### 2.3 qRT‐PCR assays

For miRNA detection, the input RNAs were reverse transcribed (RT) in a small‐scale reaction by the TaqMan miRNA Reverse Transcription Kit (Applied BioSystems, Foster City, CA, USA) following the manufacturer's protocol (5 μL total volume with 1 μL of input RNA; components other than the input RNA were prepared as a large‐volume master mix). RT products were diluted 1:5 and subjected to qPCR in triplicate using the TaqMan miRNA Assay Kit (Applied BioSystems) according to the manufacturer's protocol in a small‐scale reaction (10 μL total volume with 4.5 μL of diluted RT products; components other than input RT products were prepared as a large‐volume master mix). miRNA expression levels were normalized to U6 and calculated using the equation 2−ΔCt, where ΔCt=cycle threshold (Ct) (miRNA)—Ct (U6).

For the detection of gene expression, first strand cDNA was synthesized from equal amounts of RNA using the Super Script TM First‐Strand Synthesis System (Life Technologies, Carlsbad, CA, USA). Quantitative PCR was performed in triplicate with the 7500 Real‐time PCR System (Applied BioSystems) using SYBR Premix Ex TaqTM (Takara, Dalian, China) to detect gene expressions of cells. PCR amplification was performed with the sets of primers designed by Primer 5.0. The primers for genes were as follows: beta‐actin (F: 5′‐CCTGGCACCCAGCACAAT‐3′; R: 5′‐GCCGATCCACACGGAGTACT‐3′) and SYK (F: 5′‐TTTTGGAGGCCGTCCACAAC‐3′; R: 5′‐ATGGGTAGGGCTTCTCTCTG‐3′). To detect the gene expression of SYK in the whole blood of people, the TaqMan assays (Assay ID for SYK: Hs00895377_m1; assay ID for beta‐actin: Hs01060665_g1 from Life Technologies) were applied. Gene expression levels were calculated using the comparative quantitative method (the −ΔΔCT method) and normalized by beta‐actin.

### 2.4 Target prediction and miRNA selection

The miRNAs that might target SYK gene were firstly predicted by in silico analysis using a bioinformatics tool: TargetScan (http://www.targetscan.org/vert_71/). We previously explored the miRNA expression profiles of 25 IS patients and 25 control volunteers using miRNA microarray and found that 455 miRNAs were differentially expressed (unpublished data, *P* < 0.05). Ultimately, a total of 20 differentially expressed miRNAs with the most negative cumulative weighted context++ score by TargetScan Human 7.1 were selected for further investigation as shown in Table S1.

### 2.5 Cell culture and chemicals

Cells lines of the THP‐1, U937, HEK293T (human embryonic kidney) and HUVEC (human umbilical vein endothelial cell) were purchased from the Procell company (Wuhan, China). THP‐1, U937 and HUVECs were cultured in RPMI‐1640 medium (Gibco, Grand Island, NY, USA), and HEK293T cells were maintained in Dulbecco's modified Eagle's medium supplemented with 10% foetal calf serum (Gibco) and penicillin/streptomycin in a humidified atmosphere containing 5% CO_2_ at 37°C. For THP‐1 and U937 cells, approximately 50 nM miRNA mimic or control mimic purchased from RIBObio (RIBObio company, Guangzhou, Guangdong Province, China) was transfected into cells for 48 hours using the riboFECT CP transfection kit (Guangzhou RIBObio company) according to the manufacturer's protocol. All experiments were conducted with cells at a logarithmic growth stage.

### 2.6 Protein extraction and western blot analysis

Cells were harvested in RIPA lysis buffer (Beyotime, Shanghai, China) containing 1 μM PMSF (Sigma‐Aldrich, St. Louis, MO, USA) and a Protease Inhibitor Mixture (Roche Diagnostics, Shanghai, China). Proteins were separated by SDS‐PAGE, transferred to nitrocellulose membranes. After blocking with 5% non‐fat milk for 1 hour at room temperature, the membranes were incubated with rabbit anti‐SYK (13198S; Cell Signaling Technology, Danvers, MA, USA) or mouse anti‐beta actin (A5316; Sigma‐Aldrich) primary antibody overnight at 4°C, followed by an incubation with goat anti‐rabbit (111‐035‐003; Jackson Immunoresearch, West Grove, PA, USA) or goat antimouse (#6170‐05; Southern Biotech, Birmingham, AL, USA) IgG secondary antibodies conjugated with horseradish peroxidase at room temperature for 2 hours. Proteins were visualized by an enhanced chemiluminescences detection kit (Amersham Bioscience, Buckinghamshire, UK).

### 2.7 Cloning of 3′UTR of SYK mRNA and luciferase reporter assay

The 3′UTR sequences of SYK were ligated into the Psicheck2 luciferase reporter vector (Promega, Madison, WI, USA). HEK293T cells were seeded into 96‐well plates at 5000 cells/well and incubated at 37°C for 24 hours. About 100 ng luciferase constructs and 50 nM miRNA mimics were transfected into each well using riboFECT CP transfection kit (Guangzhou RIBObio company) following the manufacturer's protocol. We also performed the luciferase reporter assay in HUVECs. HUVECs were seeded into 96‐well plates at 1 × 10^4^ cells/well and incubated at 37°C for 24 hours. About 100 ng luciferase constructs and 50 nM miRNA mimics were transfected into each well using Lipofectamine 3000 (Invitrogen) according to the manufacturer's protocol. At 24 hours post transfection, cells were lysed and luciferase assays were performed with a Dual‐Luciferase reporter assay system (Promega) following the manufacturer's protocol on a single automatic injection Mithras luminometer (Berthold Technologies, Bad Wildbad, Germany). Ratios of firefly luciferase readings to Renilla luciferase readings were calculated.

### 2.8 Statistical analysis

The normal distribution of data was tested using the one‐sample Kolmogorov‐Smirnov test. Continuous variables were expressed as the mean ± SD or median (25th–75th quartile), and categorical variables were expressed as frequency. Differences in clinical characteristics between cases and controls were examined by the chi‐squared test for categorical variables, by Student's *t*‐test for normally distributed data, or by the Mann‐Whitney *U* test for skewed data. The difference in miR‐129‐2‐3p expression levels between cases and controls, and SYK mRNA or protein levels between cells with different treatment was examined by Student's *t*‐test. Comparison of the relative luciferase activities (RLAs) between cell groups were examined by one‐way ANOVA. The odds ratios (ORs) and 95% confidence intervals (CIs) were calculated to assess the association between miR‐129‐2‐3p and IS using the multivariate logistic regression model after adjusting for conventional risk factors, including age, sex, smoking, drinking, hypertension and diabetes mellitus. Correlations between miRNA expression and clinical variables were analysed using the Pearson or Spearman correlation test. All statistical were performed with SPSS 16.0 software (Statistical Package for the Social Sciences, Chicago, IL, USA). A value of *P* < 0.05 was considered significant (two‐tailed).

## 3 RESULTS

### 3.1 Regulation of SYK expression by miR‐129‐2‐3p

**Figure 1:**
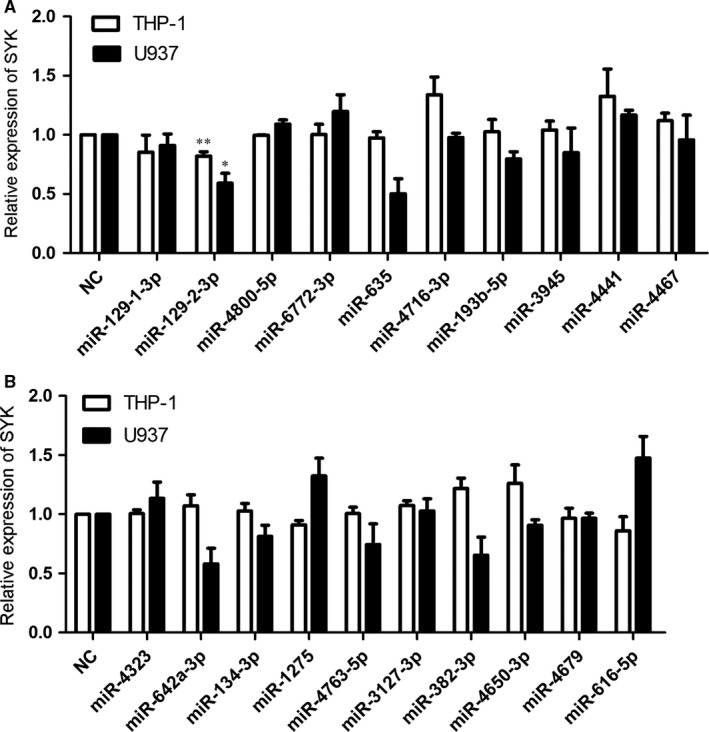
Relative mRNA levels of the gene SYK in cells transfected with miRNA mimics. (A and B) THP‐1 or U937 cells were transfected with 50 nM mimic control or the miRNA mimics, respectively. After 48 hours of transfection, cells were harvested and the total RNA was extracted. The group of cells treated with mimic control (NC) served as the control, and the relative expression of SYK was detected by qRT‐PCR, normalized to the mRNA levels of beta‐actin in each group. Quantifications of the mRNA levels of SYK were expressed as mean ± SD from three independent experiments. **P < 0.01, *P < 0.05 compared with the control by the Student's *t‐*test

**Figure 2:**
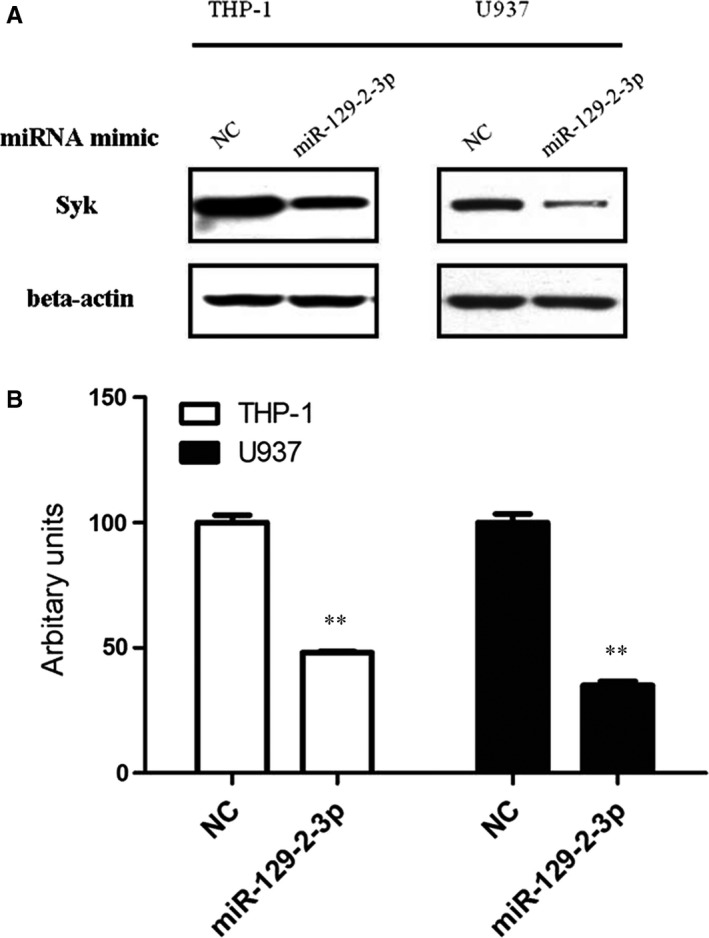
The relative protein levels of gene SYK in cells transfected with miRNA mimics. (A) THP‐1 or U937 cells were transfected with 50 nM mimic control or the miR‐129‐2‐3p mimic, respectively. After 48 hours of transfection, cells were harvested and the total protein lysates were analysed for Syk protein levels by Western blotting. Beta‐actin served as the loading control. (B) Quantifications of immunoblotting were expressed as mean ± SD from three independent experiments. **P < 0.01 compared with the control group by the Student's *t*‐test

The microarray data and the cumulative weighted context++ scores of the 20 miRNAs were shown in Table S1. Among the miRNAs, after transfection of miR‐129‐2‐3p mimics for 48 hours, the mRNA level of SYK gene was both down‐regulated in THP‐1 and U937 cells, with a decreased level to 90% (THP‐1) and 55% (U937) of the cells, respectively, compared with the mimic control (Figure 1A). However, the mRNA levels of SYK gene showed no alteration in cells transfected with other miRNA mimics (Figure 1A and B). Afterwards, we evaluated the change in SYK protein levels after miR‐129‐2‐3p transfection. Results suggest that cells transfected with miR‐129‐2‐3p mimics showed decreased protein levels of SYK in both THP‐1 (to a 50% extent) and U937 cells (to a 40% extent), as shown in Figure 2A and B.

### 3.2 Direct binding between miR‐129‐2‐3p and SYK gene

**Figure 3:**
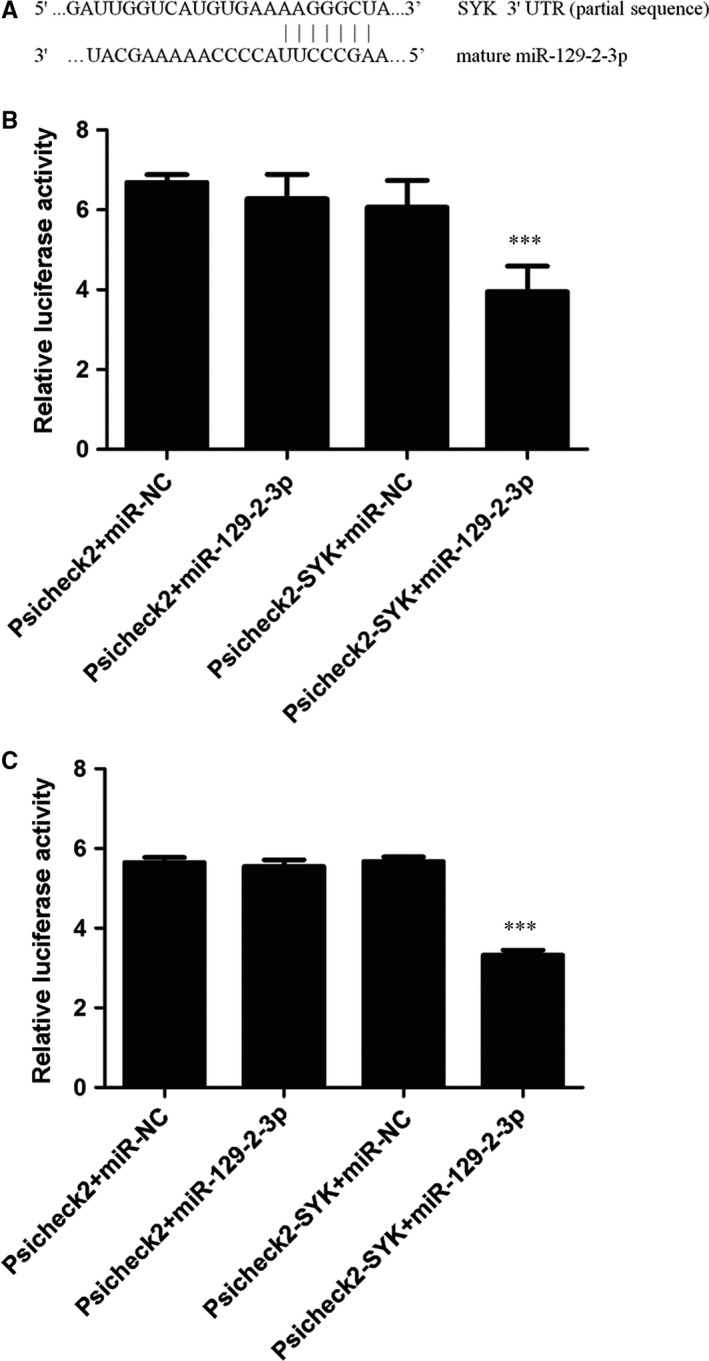
Direct binding between miR‐129‐2‐3p and the 3′UTR of gene SYK. (A) in silico prediction of miR‐129‐2‐3p and *SYK* mRNA interactions showed the potential targeting sites on the 3′UTR of *SYK* gene. The HEK293T cells (B) and HUVECs (C) were cotransfected with luciferase constructs and mimic control/miR‐129‐2‐3p respectively. After transfection for 24 hours, the relative luciferase activity was analysed by Dual‐luciferase reporter system. Data were represented as mean ± SD from three independent experiments. ***P < 0.001 compared with the control groups analysed by one‐way ANOVA

Bioinformatics assays with TargetScan 7.1 suggested that the 3′UTR of SYK gene created a putative binding site for miR‐129‐2‐3p (Figure 3A). We transfected miRNA mimics and the luciferase reporter vector Psicheck2 constructs encompassing the 3′UTR fragment of *SYK* to HEK293T cells and HUVECs, respectively, and the RLAs were measured. Results suggested that no significant change in RLA was found between the three control cell groups (Psicheck2 + miR‐NC, Psicheck2 + miR‐129‐2‐3p, and Psicheck2‐*SYK*+miR‐NC). However, the RLA of 293T cells transfected with miR‐129‐2‐3p and Psicheck2‐*SYK* decreased by about 33% compared with the cells transfected with Psicheck2‐*SYK* and mimic control (Figure 3B), suggesting that *SYK* might be a direct target of miR‐129‐2‐3p. Similar results were found in the HUVECs. The RLA of cells transfected with miR‐129‐2‐3p and Psicheck2‐*SYK* decreased by about 41% compared with the cells transfected with Psicheck2‐*SYK* and mimic control (Figure 3C).

### 3.3 General characteristics of the study population

The general characteristics of the case‐control population are shown in Table 1. The levels of fasting glucose, total cholesterol, mean platelet volume (MPV) and plateletcrit were all significantly higher in IS patients compared with the control volunteers while the level of high‐density lipoprotein cholesterol in IS patients was significantly lower compared with the control volunteers (*P* < 0.05). Smoking frequency, history of hypertension or diabetes mellitus were all significantly higher in patients (*P* < 0.05), while the drinking frequency of IS patients was significantly lower than control volunteers (*P* = 0.001). The other variables showed no difference between the two groups. The median onset time from stroke onset to hospital admission was 48 hours, and the average score of National Institute of Health stroke scale was 5 for the patient cohort. Moreover, about 55 patients showed formation of carotid atherosclerotic plaque.

**Table 1:** General characteristics of the study population

Variables	Control (n = 270)	Stroke (n = 270)	*P* value
Age, year	55.87 ± 10.42	57.00 ± 10.20	0.214^a^
Male, n (%)	158 (58.5)	160 (59.3)	0.861^b^
FG, mmol/L	5.29 (4.93, 5.87)	5.52 (4.97, 6.77)	<0.003^c^
TC, mmol/L	4.84 ± 1.38	5.07 ± 1.25	0.048^a^
TG, mmol/L	1.38 (0.92, 2.30)	1.32 (0.96, 1.83)	0.168^c^
HDL‐c, mmol/L	1.26 ± 0.31	1.05 ± 0.27	<0.001^a^
LDL‐c, mmol/L	3.10 ± 0.86	3.03 ± 0.95	0.384^a^
PLT (×10^9^/L)	229 ± 52	228 ± 98	0.842^a^
PDW, %	16.00 (15.70, 16.43)	16.10 (13.83, 16.60)	0.894^c^
MPV, fL	8.60 ± 1.35	9.09 ± 1.48	<0.001^a^
PCT	0.19 ± 0.05	0.21 ± 0.09	0.041^a^
Smoking, n (%)	39 (14.4)	63 (23.3)	0.008^b^
Drinking, n (%)	48 (17.8)	23 (8.5)	0.001^b^
Hypertension, n (%)	59 (21.9)	158 (58.5)	<0.001^b^
Diabetes mellitus, n (%)	17 (6.3)	45 (16.7)	<0.001^b^
Onset time, hour	‐	48 (13, 72)	‐
NIHSS	‐	5 (2, 13)	‐
Atherosclerotic plaque, n (%)	‐	55 (20.4)	‐

Data are expressed as mean ± SD, median (25th, 75th quartiles) or percentages.

FG, fasting glucose; TC, total cholesterol; TG, triglycerides; HDL‐C, high‐density lipoprotein cholesterol; LDL‐C, low‐density lipoprotein cholesterol; PLT, blood platelet; PDW, platelet distribution width; MPV, mean platelet volume; PCT, plateletcrit; NIHSS, National Institute of Health stroke scale.

a Student's *t*‐test for the difference between ischaemic stroke patients and controls.

b Chi‐square test for the difference in the distribution frequencies between ischaemic stroke patients and controls.

c Mann‐Whitney *U* test for the differences between ischaemic stroke patients and controls.

### 3.4 Association of miR‐129‐2‐3p expression with the occurrence of IS

**Figure 4:**
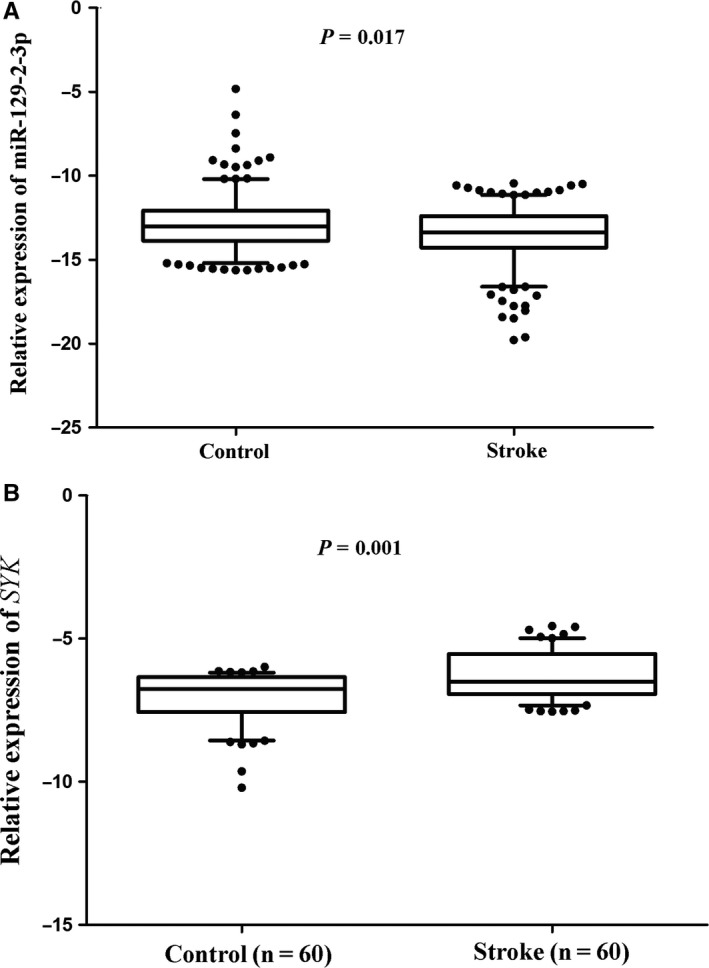
The expression levels of miR‐129‐2‐3p and SYK gene in IS patients and control volunteers. (A) miR‐129‐2‐3p expression was detected in the whole blood of 270 ischaemic stroke patients and 270 control volunteers. The relative expression levels were normalized to U6 and then log‐transformed. (B) The expression levels of SYK gene were detected in the whole blood of 60 ischaemic stroke patients and 60 control volunteers. The relative expression levels were normalized to beta‐actin and then log‐transformed. The whiskers of the plots represent the 5‐95 percentiles. Comparison of gene expression between the two groups of cohorts was analysed by the Student's *t* test

The miR‐129‐2‐3p expression levels were detected in the blood cells of 270 control volunteers and 270 IS patients. As shown in Figure 4A, results suggested that the expression level of miR‐129‐2‐3p was significantly lower in IS patients compared with the control volunteers (log‐transformed expression levels relative to U6, −13.00 ± 1.68 vs −13.35 ± 1.75, *P* = 0.017). Association of miR‐129‐2‐3p expression with the occurrence of IS was analysed by multivariate logistic regression analysis. Results showed that the blood level of miR‐129‐2‐3p was negatively associated with the risk of IS (**Model I**: adjusted OR: 0.88; 95% CI: 0.80‐0.98; *P* = 0.021; Table 2) after adjusting for conventional risk factors, including age, sex, smoking, drinking and history of diabetes mellitus. However, when the variable of hypertension history was added to the model I, the association between miR‐129‐2‐3p expression and the occurrence of IS was missing (**Model II**: adjusted OR: 0.92; 95% CI: 0.82‐1.02; *P* = 0.119).

**Table 2:** Association of the expression level of miR‐129‐2‐3p with ischaemic stroke

Variables	Adjusted OR (95% CI)			
	Model I^a^	*P*‐value	Model II^b^	*P*‐value
Age	1.01 (0.99, 1.03)	0.136	0.99 (0.98, 1.02)	0.759
Sex	1.10 (0.74, 1.62)	0.642	0.99 (0.65, 1.51)	0.969
Smoking	2.52 (1.49, 4.25)	0.001	2.98 (1.70, 5.20)	<0.001
Drinking	0.30 (0.17, 0.55)	<0.001	0.30 (0.16, 0.56)	<0.001
Hypertension	‐	‐	5.00 (3.33, 7.52)	<0.001
Diabetes mellitus	2.84 (1.56, 5.16)	0.001	1.88 (0.99, 3.58)	0.053
miR‐129‐2‐3p	0.88 (0.80, 0.98)	0.021	0.92 (0.82, 1.02)	0.119

OR, odds ratio; CI, confidence interval.

a Adjusted for age, sex, diabetes mellitus, smoking and drinking by logistic regression analysis.

b Adjusted for age, sex, hypertension, diabetes mellitus, smoking and drinking by logistic regression analysis.

### 3.5 SYK expression in the blood cells of IS patients

To figure out the potential negative regulation of *SYK* by miR‐129‐2‐3p, we further detected the mRNA levels of *SYK* in the blood cells of 60 IS patients and 60 control volunteers. Results suggested that the relative expression levels of *SYK* were significantly higher in IS patients compared with the control volunteers (log‐transformed expression levels relative to beta‐actin, −6.29 ± 0.85 vs −7.06 ± 0.92, *P* = 0.001), as shown in Figure 4B.

### 3.6 Correlation between miR‐129‐2‐3p expression and clinical parameters

**Figure 5:**
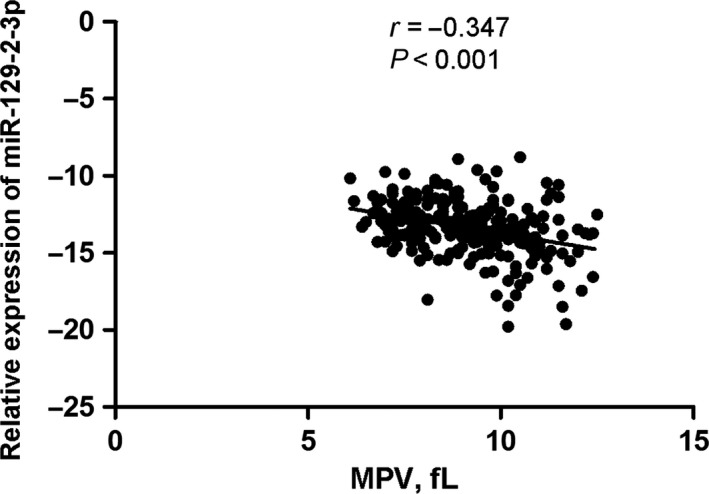
Correlation between miR‐129‐2‐3p expression and the platelet parameter. Correlation between miR‐129‐2‐3p expression and the mean platelet volume was analysed by Pearson's correlation test in stroke patients

Results of the correlation analysis between miR‐129‐2‐3p expression and the clinical parameters suggest that the expression levels of miR‐129‐2‐3p in blood cells were negatively correlated with the MPV level (*r* = −0.347, *P* < 0.001) in stroke patients, as shown in Figure 5.

## 4 DISCUSSION

Combining bioinformatics prediction with target gene expression analysis, we found in this study that miR‐129‐2‐3p may be involved in the pathogenesis of IS by regulating the expression of SYK gene, which is a critical regulator in the development of atherosclerosis. The whole blood level of miR‐129‐2‐3p was significantly lower in IS patients compared with control volunteers in a Chinese population, and the higher level of miR‐129‐2‐3p was associated with a decreased occurrence of IS. Moreover, the expression of SYK gene in the blood cells of patients was significantly higher compared with control volunteers. The biological consequence of altered expression of miR‐129‐2‐3p and SYK in stroke warrants further investigation.

The motive that initiates the conduction of this study was the association that we found between SYK gene polymorphism and the risk of IS, which was identified by exome sequencing in one of our previous studies,^11^ while not yet published. In recent years, studies about the role of SYK gene in stroke have attracted great research interest. By transient middle cerebral artery occlusion rat model, Boddaert et al demonstrated an obvious up‐regulation of CD8 along with SYK transducer in dissected perilesional brain tissue, and suggested that increased CD8 signaling in the post‐stroke brain was primarily associated with microglia/macrophages and could independently drive M1 polarization.^22^ Gotru et al demonstrated that the altered phosphorylation of Syk accounted for the global platelet activation defect in response to stimulation of the major platelet receptor glycoprotein VI (GPVI) in mice with deleted kinase activity of transient receptor potential cation channel, subfamily M, member 7 (TRPM7).^23^ These defects translated into an impaired platelet aggregate formation under flow and protection of the mice from arterial thrombosis and IS in vivo.^23^ Syk is a major signaling node for collagen receptor GPVI, which couples to the FcRγ chain and its immune‐receptor tyrosine activation motif to initiate platelet activation. In contrast, Syk has no, or perhaps a limited role in response to G‐protein coupled receptors, such as those for thrombin, thromboxane A2, and adenosine diphosphate. These are the targets of conventional antiplatelet therapies with limitations in efficacy and safety. Therefore, the promise for the research and development of Syk inhibitor relies on the fact that the thrombosis is curtailed while haemostasis is preserved.^24^ Proof of concept of the benefits of Syk inhibition in mouse models of thrombosis has been reported using small molecules^25^, ^26^ or miRNA approach.^27^ Van Eeuwijk, Nieswandt and colleagues reported a comprehensive approach to their proof of concept for the action of BI1002494 in IS. Results suggested that not only did pretreatment with BI1002494 reduce infract size without bleeding, but also administration of the inhibitor after the stroke was generated had similar benefits, providing a compelling case for continued investigation and translation of SYK inhibition strategy to human patients.^28^ Inspired by our previous findings and the protective role of SYK inhibition in atherosclerosis‐related diseases, we have been suggested that SYK inhibition might be partially regulated by miRNAs.

Alteration of SYK expression after miRNA transfection, and the results of luciferase activity experiments revealed that SYK gene might be a direct target of miR‐129‐2‐3p. The opposite direction of change in SYK and miR‐129‐2‐3p expression in stroke further validated our hypothesis that SYK expression might be negatively regulated by miR‐129‐2‐3p. Moreover, our study is the first to describe that the expression level of miR‐129‐2‐3p was negatively associated with the occurrence of IS. The previous studies about miR‐129‐2‐3p mainly focused on cancer. For instance, Cui et al found that methylation‐mediated miR‐129‐2‐3p down‐regulation could promote epithelial‐mesenchymal transition, leading to in vitro invasion and in vivo metastasis of hepatocelluar cancer cells.^29^ Tsai et al demonstrated that the expression of miR‐129‐2‐3p was down‐regulated by DNA hyper‐methylation in primary gastric cancers, and the low expression was associated with poor clinical pathological features.^30^ For cardiovascular diseases, Cakmak et al demonstrated that the sera level of miR‐129‐2‐3p was decreased in patients with chronic congestive heart failure.^31^ Collectively, the expression of miR‐129‐2‐3p seems to be universally down‐regulated in the pathological conditions, no matter in cancer or in cardiovascular diseases, suggesting a critical role of miR‐129‐2‐3p in many pathological conditions. However, the role of miR‐129‐2‐3p in stroke has been rarely reported. Interestingly, as we were preparing this manuscript, Tsai et al found that osteopontin could promote monocyte migration by up‐regulating IL‐17 expression through miR‐129‐2‐3p inhibition in osteoblast in rheumatoid arthritis disease. Notably, the Syk‐dependent PI3K/Akt pathway also played a key role in osteopontin‐induced increase in monocyte migration and the decrease in miR‐129‐2‐3p expression.^32^ In the context of that, as we found in this study that SYK expression could also be regulated by miR‐129‐2‐3p, it seems that there might be a reciprocal regulation circle between miR‐129‐2‐3p and Syk, which needs further investigation.

Platelets are small cell fragments in the blood. The classical function of platelets is coverage and closure of endothelial wounds, and contact between platelets and the subendothelial matrix triggers their activation and drives thrombus formation. Platelet activation can confer pro‐atherosclerotic effects, and plays critical roles in the development of atherosclerosis and cardiovascular diseases.^33^ MPV is a useful biomarker for platelet activity. O'Malley et al noticed an increase in MPV in all subtypes of IS.^34^ This increase was already detectable in the acute phase (within 48 hours of the onset of symptoms) and persisted long after the stroke (the second measurement was performed after 6 months). This finding led to the conclusion that increased MPV values were probably pre‐existent to the acute event, and were potentially involved in the genesis of the disease, considering, in particular, that mean platelet life is only 8 days. Besides, MPV can even provide some information about the prognosis of stroke patients.^35^ In this study, we found that the MPV levels of IS patients were significantly higher than the control volunteers and the decreased level of miR‐129‐2‐3p was negatively correlated with MPV in the stroke patients (Figure 5). Several platelet functions rely on SYK signaling. GpVI, the major collagen receptor of platelets, is an FcRγ‐associated receptor closely related to FcαRs, which may explain that GpVI, like other FcR family members, signals through SYK.^36^ The C‐type lectin CLEC2 has also been shown to signal through SYK in platelets.^37^ Taken together, we conclude that the underlying mechanism for miR‐129‐2‐3p in IS may involve activation of the platelet function by regulating the Syk‐GpVI signaling pathway, which needs further investigations about the underlying mechanism.

This study has several strengths. First, the expression level of miR‐129‐2‐3p was detected in a case‐control population with comparatively large sample size, providing sufficient statistical power for multivariate analysis. We calculated the statistical power using the tool PASS. This study has 86% power to detect convincing association with P0 = 0.5, α = 0.05, OR = 1.5, N = 540. Further studies with larger sample size are required to confirm the role of miR‐129‐2‐3p in IS. Second, we tested miRNA‐gene regulation by conducting bioinformatics prediction, cell experiments and detecting the RLAs to show the direct binding between miRNA and the target gene, which provides a plausible biological explanation for the epidemiological findings in our study. Third, the correlation between miR‐129‐2‐3p expression and the platelet parameters further indicated the potential functional role of miR‐129‐2‐3p in platelet activation. However, several limitations should also be addressed. First, because this is a case‐control study, potential selection bias may influence the interpretation of the results. Second, the biological consequence involved in platelet activation following changes in miRNA or SYK gene expression also merits further investigation. Last, emerging evidence show that the expression quantitative trait locus (eQTLs) describe variants associated with changes in the transcriptome, either acting in a cell‐type specific manner or nearby transcripts (cis) or on transcripts being further away in the genome (trans), especially in the research field of cancer.^38^, ^39^ Although challenges and opportunities both exist in stroke genetics,^40^ accumulating evidence to explore the functional SNPs associated with stroke have been reported recently, by means of eQTLs.^41^, ^42^ Inspired by the above studies, we will further combine in silico and experimental approaches to explore the functional SNPs of SYK gene, which might improve our understanding of disease mechanism in stroke and likely provide the field with novel targets and pathways for intervention.

In summary, this study suggests that the blood cell level of miR‐129‐2‐3p is significantly lower in IS patients and associated with the occurrence of IS. SYK gene might be a direct target of miR‐129‐2‐3p, and the expression levels of SYK gene were significantly higher in IS patients compared with control volunteers. In addition, miR‐129‐2‐3p expression was negatively correlated with the platelet parameter MPV. Further investigations are needed to explore the biological relevance of our findings.

## CONFLICT OF INTEREST

The authors confirm that there are no conflicts of interest.

## AUTHOR CONTRIBUTION

SLH, ZQL and JQC conceived and designed the research study. SLH, ZQL, YW, YZW and CHX participated in the experiments and drafted the manuscript. YZW, YWZ, QHX, YG and YSG contributed to the sample collection and interpretation the data. YW, LL and DL performed the statistical analysis. YBK, LZ, YG and JQC revised the manuscript. All authors read and approved the final manuscript.
