# Supplementary material for: miR‐129‐2‐3p directly targets SYK gene and associates with the risk of ischaemic stroke in a Chinese population
Source: J Cell Mol Med. 2018 Nov 29;23(1):167–76. doi: 10.1111/jcmm.13901 (PMC6307781; doi:10.1111/jcmm.13901)
Supplement: Supplementary file 1 [file JCMM-23-167-s001.docx]

**Supplementary Table 1. The negative cumulative weighted context++ score and microarray data of the 20 miRNAs**

| **miRNA** | **Weighted context++ score** | **Control** | | |  | **Stroke** | | |  | **Fold change** | **P value*** |
| --- | --- | --- | --- | --- | --- | --- | --- | --- | --- | --- | --- |
|  |  | **Mean of copy** | **Mean**  **(normalized copy)** | **CV** |  | **Mean of copy** | **Mean**  **(normalized copy)** | **CV** |  |  |  |
| hsa-miR-129-1-3p | -0.39 | 51.68 | 3.62 | 0.40 |  | 55.44 | 5.44 | 0.24 |  | 3.54 | <0.001 |
| hsa-miR-129-2-3p | -0.39 | 21.78 | 3.24 | 0.69 |  | 121.74 | 5.58 | 0.32 |  | 5.08 | <0.001 |
| hsa-miR-4800-5p | -0.27 | 213.66 | 4.86 | 0.23 |  | 114.16 | 7.62 | 0.10 |  | 6.79 | <0.001 |
| hsa-miR-6772-3p | -0.26 | 50.8 | 3.39 | 0.61 |  | 140.50 | 6.86 | 0.13 |  | 11.05 | <0.001 |
| hsa-miR-635 | -0.24 | 80.58 | 4.33 | 0.31 |  | 70.46 | 5.89 | 0.21 |  | 2.94 | <0.001 |
| hsa-miR-4716-3p | -0.22 | 30.16 | 2.39 | 0.40 |  | 13.60 | 3.48 | 0.39 |  | 2.12 | 0.002 |
| hsa-miR-193b-5p | -0.21 | 11.48 | 2.24 | 0.31 |  | 42.70 | 5.22 | 0.22 |  | 7.89 | <0.001 |
| hsa-miR-3945 | -0.21 | -14.7 | 1.54 | 0.54 |  | 47.74 | 5.01 | 0.22 |  | 11.08 | <0.001 |
| hsa-miR-4441 | -0.19 | 232.56 | 6.82 | 0.19 |  | 309.14 | 8.36 | 0.11 |  | 2.91 | <0.001 |
| hsa-miR-4467 | -0.18 | 6158.14 | 12.39 | 0.06 |  | 14207.02 | 13.60 | 0.04 |  | 2.32 | <0.001 |
| hsa-miR-4323 | -0.17 | 58.72 | 3.73 | 0.48 |  | 65.46 | 5.49 | 0.29 |  | 3.37 | <0.001 |
| hsa-miR-642a-3p | -0.09 | 106.58 | 3.83 | 0.50 |  | 165.86 | 7.34 | 0.18 |  | 11.43 | <0.001 |
| hsa-miR-134-3p | -0.08 | 204.12 | 5.45 | 0.27 |  | 321.00 | 9.08 | 0.13 |  | 12.43 | <0.001 |
| hsa-miR-1275 | -0.05 | 606.78 | 8.43 | 0.11 |  | 776.98 | 9.91 | 0.08 |  | 2.77 | <0.001 |
| hsa-miR-4763-5p | -0.05 | 35.8 | 3.72 | 0.51 |  | 171.98 | 5.22 | 0.43 |  | 2.82 | 0.015 |
| hsa-miR-3127-3p | -0.03 | 57.7 | 3.17 | 0.71 |  | 54.44 | 5.19 | 0.35 |  | 4.05 | 0.001 |
| hsa-miR-382-5p | -0.02 | 24.36 | 3.26 | 0.36 |  | 115.42 | 6.31 | 0.21 |  | 8.24 | <0.001 |
| hsa-miR-4650-3p | -0.02 | 78.14 | 3.47 | 0.31 |  | 117.98 | 7.14 | 0.19 |  | 12.75 | <0.001 |
| hsa-miR-4679 | -0.02 | 226.56 | 4.42 | 0.40 |  | 74.90 | 5.91 | 0.37 |  | 2.81 | 0.011 |
| hsa-miR-616-3p | -0.02 | -28.36 | 1.44 | 0.38 |  | 37.74 | 4.00 | 0.34 |  | 5.89 | <0.001 |

CV, coefficient of variation; Fold change=2^(normalized mean copy of stroke)/ 2^(normalized mean copy of control).

*Comparison of miRNA expressions by Student’s *t* test.
